# Methodological considerations on near-infrared spectroscopy derived muscle oxidative capacity

**DOI:** 10.1007/s00421-024-05421-6

**Published:** 2024-02-24

**Authors:** Letizia Rasica, Erin Calaine Inglis, Raffaele Mazzolari, Danilo Iannetta, Juan M. Murias

**Affiliations:** 1 Faculty of Kinesiology, University of Calgary, Calgary, Canada; 2 Department of Sport Science, University of Innsbruck, Innsbruck, Austria; 3 College of Health and Life Sciences, Hamad Bin Khalifa University, Doha, Qatar

**Keywords:** NIRS, Skeletal muscle oxidative metabolism, Fitness level, Vastus lateralis, Tibialis anterior

## Abstract

**Purpose:**

Different strategies for near-infrared spectroscopy (NIRS)-derived muscle oxidative capacity assessment have been reported. This study compared and evaluated (I) approaches for averaging trials; (II) NIRS signals and blood volume correction equations; (III) the assessment of vastus lateralis (VL) and tibialis anterior (TA) muscles in two fitness levels groups.

**Methods:**

Thirty-six participants [18 chronically trained (CT: 14 males, 4 females) and 18 untrained (UT: 10 males, 8 females)] participated in this study. Two trials of twenty transient arterial occlusions were performed for NIRS-derived muscle oxidative capacity assessment. Muscle oxygen consumption ($\dot{V}$O_2_*m*) was estimated from deoxygenated hemoglobin (HHb), corrected for blood volume changes following Ryan (HHbR) and Beever (HHbB) equations, and from oxygen saturation (StO_2_) in VL and TA.

**Results:**

Superimposing or averaging $\dot{V}$O_2_*m* or averaging the rate constants (*k*) from the two trials resulted in equivalent *k* values [two one-sided tests (TOST) procedure with 5% equivalence margin—*P* < 0.001]. Whereas HHbR (2.35 ± 0.61 min^−1^) and HHbB (2.34 ± 0.58 min^−1^) derived *k* were equivalent (*P* < 0.001), StO_2_ derived *k* (2.81 ± 0.92 min^−1^) was greater (*P* < 0.001) than both. *k* values were greater in CT *vs* UT in both muscles (VL: + 0.68 min^−1^, *P* = 0.002; TA: + 0.43 min^−1^, *P* = 0.01).

**Conclusion:**

Different approaches for averaging trials lead to similar *k*. HHb and StO_2_ signals provided different *k*, although different blood volume corrections did not impact *k*. Group differences in *k* were detected in both muscles.

**Supplementary Information:**

The online version contains supplementary material available at 10.1007/s00421-024-05421-6.

## Introduction

The skeletal muscle oxidative capacity is the ability of the muscle to utilize oxygen for adenosine triphosphate (ATP) resynthesis and it is directly linked to mitochondrial respiration (Holloszy 1967).

While exercise training can positively influence mass-specific mitochondrial respiration, affecting both mitochondrial protein synthesis and mitochondrial content (Granata et al. 2018), physical inactivity, aging, and/or chronic disease can lead to sub-optimal mitochondrial adaptations that impair ATP resynthesis (McCully et al. 2011; Buso et al. 2019; Grevendonk et al. 2021).

Therefore, the evaluation of muscle oxidative capacity is of utmost interest to characterize exercise performance and exercise tolerance across the age, fitness, and health spectrums (Ryan et al. 2013b; Willingham and McCully 2017). In the past, the evaluation of mitochondrial respiration has been limited to invasive or costly assessments (biopsy or 31P-MRS); however, within the last ~ 10 years, the investigation of skeletal muscle oxidative capacity through near-infrared spectroscopy (NIRS) has gained popularity in both research and clinical settings (Adami and Rossiter 2018).

NIRS is a noninvasive tool that can be used to gain insights into skeletal muscle oxidative capacity. NIRS light interrogates the underly tissue providing data related to the changes in the balance between oxygen delivery and utilization (Grassi and Quaresima 2016). Based on a concept initially proposed by Hamaoka and colleagues (Hamaoka et al. 1996; Motobe et al. 2004), NIRS evaluation of muscle oxidative capacity has been utilized by different research groups and it has subsequently been used to interpret relative differences across a wide range of ages, fitness levels, and clinical conditions (e.g., Ryan et al. 2013b; Adami et al. 2017; Lagerwaard et al. 2020). It has been recently demonstrated that NIRS-derived muscle oxidative capacity can by itself explain cycling endurance performance better than predictive theoretical models combining traditional variables of aerobic performance in endurance trained individuals (Batterson et al. 2020).

The technique consists of active or electrically-induced muscle contractions, followed by brief and sequential ischemic periods during which the rate of increase in concentration of deoxygenated hemoglobin (HHb) or the rate of decrease in the oxygen saturation signal (StO_2_) is determined to subsequently estimate muscle oxygen consumption ($$\dot{V}$$O_2_*m*) with a selected temporal resolution (Ryan et al. 2012; Adami et al. 2017). These $$\dot{V}$$O_2_*m* values allow for the identification of the $$\dot{V}$$O_2_*m* off-kinetics (i.e., the rate of change in $$\dot{V}$$O_2_*m* from exercise back to resting values), which follows an exponential time course. The exponential decay rate constant of $$\dot{V}$$O_2_*m* (*k*) and/or time constant (τ $$)$$ are indicative of muscle oxidative capacity, with faster $$\dot{V}$$O_2_*m* kinetics described by greater *k* and/or smaller τ values (Zuccarelli et al. 2021). This NIRS-derived approach for evaluating muscle oxidative capacity has been validated by studies where the kinetics of recovery of $$\dot{V}$$O_2_*m* has been shown to be well correlated with the kinetics of recovery of [PCr], determined by 31P-MRS following exercise (Ryan et al. 2013c), and the maximal ADP-stimulated mitochondrial respiration, evaluated by high-resolution respirometry of permeabilized skeletal muscle fibers (Ryan et al. 2014).

Due to the growing popularity of the NIRS technique, different approaches have been used by several research groups in terms of (I) modality (Southern et al. 2014), intensity (Ryan et al. 2013a) and duration of the exercise (Zuccarelli et al. 2020), (II) NIRS variables analyzed (Adami et al. 2017; Beever et al. 2020), and (III) muscles investigated (Hanna et al. 2021; Lagerwaard et al. 2021). In relation to this, recommendations have been formulated over the years to reduce errors and variability. These include, (I) averaging subsequent trials to improve signal-to-noise ratio, (II) correcting for changes in hemoglobin volume when using the HHb signal, (III) employing proper contractions intensities and durations to stimulate oxidative phosphorylation without limiting blood flow, and (IV) limiting the influence of adipose tissue thickness (ATT) to successfully interrogate the underlying muscles (Barstow 2019). Even though these recommendations seem appropriate, it is currently unknown whether and to what extent the method of analysis of choice impacts the quantification of muscle oxidative capacity through NIRS.

Thus, to gain clarity on how the different analysis strategies proposed in the literature might affect the evaluation of muscle oxidative capacity using NIRS-derived outcomes, this study compared different methods for averaging trials, using different NIRS signals with and without blood volume correction equations, in young healthy females and males of different fitness levels. Additionally, to overcome the possible issues associated with different ATT, this research simultaneously evaluated NIRS-derived oxidative capacity in the vastus lateralis (VL) and tibialis anterior (TA) muscles as these muscles are normally characterized by greater and lower ATT, respectively.

## Materials and methods

### Participants

A total of 36 young healthy participants [age, 24 ± 5 yr (mean ± SD); body mass, 69.1 ± 10.2 kg; height, 1.72 ± 0.09 m, body mass index, 23.3 ± 2.3 kg⋅m^−2^] volunteered for this study. All participants were healthy, non-smokers, and free of any cardiovascular or metabolic diseases contraindicating study participation. Eighteen participants (14 males, 4 females) performed their sport at a competitive level (national or international speed skaters) with ~ 15 h of training per week, mainly involving skating and cycling with minimum of 3 years of training (chronically trained, CT). The other 18 participants (10 males, 8 females) were not previously engaged in any structured exercise training program (untrained, UT).

All participants were aware of the procedures and of the possible risks associated with the experiments before giving their written informed consent to participate in the study. The protocol was approved by the Conjoint Health Research Ethics Board of the University of Calgary. All procedures were in accordance with the Declaration of Helsinki (2013) of the World Medical Association.

### Experimental design

Participants were tested on two occasions, each separated by > 48 h, in a quiet, temperature-controlled room (22–24 °C). On their first visit, participants performed an incremental exercise test for the assessment of maximal oxygen consumption ($\dot{V}$O_2max_).

On their second visit*,* participants reported to the laboratory in the morning having abstained from food (> 6 h), caffeine (> 12 h), alcohol (> 12 h), and intense exercise (24 h) before the NIRS-derived assessment of muscle oxidative capacity (see below for detailed description). Within the same visit, B-mode ultrasound (GE Logiq E9, General Electric, Yorba Linda, CA, USA) was used to determine local ATT at the site of NIRS probe placement.

### Measurements

#### Maximal oxygen consumption ($\dot{V}$O_2_max)

Participants performed an incremental exercise test to volitional fatigue on an electromagnetically-braked cycle ergometer (Velotron RacerMate Inc., Seattle, WA, USA), which varied for rate increments (20–30 W·min^−1^) depending on the fitness status of the individuals, as previously described (Inglis et al. 2021; Rasica et al. 2022).

Pulmonary ventilatory and gas exchange variables were measured using a metabolic cart with mixing chamber (Quark CPET, Cosmed, Rome, Italy). Briefly, inspired and expired flow rates were measured continuously through a low-dead space turbine which was calibrated beforehand with a 3-L syringe. Inspired and expired gases were analyzed for concentrations of O_2_ and CO_2_ after calibration with precision-analyzed gas mixtures according to the manufacturer’s specifications. In this study, a maximal effort was accepted when participants reached at least three of the following: (1) Rating of perceived exertion > 18 using the Borg’s CR20 scale (Borg 1982); (2) heart rate (HR) values > 95% of the age-predicted maximum; (3) gas exchange ratio (RER) values > 1.1; and (4) blood lactate concentration at the end of exercise (within 1 min after exercise cessation) > 8 mM. Although secondary criteria cannot be used to ascertain the achievement of $\dot{V}$O_2max_ (Poole et al. 2008), recent studies have shown that $\dot{V}$O_2max_ can be obtained from ramp incremental tests when maximal efforts are performed and without the need of the so-called verification trials (Iannetta et al. 2020). $\dot{V}$O_2max_ was computed as the greatest 30-s rolling average of $\dot{V}$O_2_ during the incremental test (Inglis et al. 2021).

#### NIRS-derived assessment of muscle oxidative capacity

To measure muscle oxidative capacity two NIRS probes (PortaMon, Artinis Medical Systems, Elst, The Netherlands) were placed respectively on the lower third of the vastus lateralis (VL) muscle (~ 10 cm above the knee joint) and on the upper third of tibialis anterior (TA) of the right leg and covered with an elastic bandage to prevent any movement and the intrusion of external light. A cuff connected to a pneumatic automatic rapid inflation system (Hokanson E20, Bellevue, WA, USA) was placed on the proximal portion of the right thigh (above the VL NIRS probe) to occlude arterial blood flow. Ankle weights (3.5 kg for UT females and 4.5 kg for UT males and CT) were placed on the ankle of the right leg. After performing 20 knee extension repetitions in a seated position at 0.5 Hz (1-s extension and 1-s flexion), the cuff was inflated to 300 mmHg for 5 s and subsequently deflated for 10 s. This inflation-deflation procedure was repeated for 20 times over a 5-min period. After 2 min of rest (7 min from the start of the previous exercise) a second trial was performed once the NIRS signals stabilized. The NIRS variables were recorded continuously throughout the repeated occlusion protocol at a frequency of 10 Hz.

$\dot{V}$O_2_*m* was estimated by calculating the slope over a 3-s span of data, excluding the data points from the first and last second of each occlusion period, to avoid any potential influenced from cuff inflation or release.

The slopes were calculated on the two NIRS variables more frequently utilized in the literature: HHb and StO_2_.

HHb data were corrected for changes in blood volume following Ryan et al. (1) (Ryan et al. 2012) and Beever et al. (2) (Beever et al. 2020) equations:

1 $$HHbR=HHb-\left(tHb \times \frac{\left|{{O}_{2}Hb}_{(t)}\right|}{\left(\left|{{O}_{2}Hb}_{(t)}\right|+\left|{HHb}_{(t)}\right|\right)}\right)$$

2 $$HHbB=\left({HHb}_{(t)}-{HHb}_{(t-0.1)}\right)-\left(1-\frac{\left|{{O}_{2}Hb}_{(t)}\right|}{\left(\left|{{O}_{2}Hb}_{(t)}\right|+\left|{HHb}_{(t)}\right|\right)}\right)\times \left({tHb}_{(t)}-{tHb}_{(t-0.1)}\right)+HHbB-0.1$$

where *t* = time (s); *tHb* = total hemoglobin; *O*_*2*_*Hb* = oxygenated hemoglobin.

Compared with the correction formula [*1*], which is the sum of the O_2_Hb and HHb signals, the equation [*2*] adjusts the instantaneous changes in O_2_Hb and HHb signals using the instantaneous change in NIRS variables (Beever et al. 2020).

To verify that the exercise modality, intensity, and duration were adequate to stimulate oxidative phosphorylation without limiting blood flow in both VL and TA, the increase in $\dot{V}$O_2_*m* during contractions was estimated from the greatest $\dot{V}$O_2_*m* recorded during the skeletal muscle oxidative capacity test, and expressed as a fold-change above the steady-state resting $\dot{V}$O_2_*m* (measured at the end of the test): a small fold-change in $\dot{V}$O_2_*m* may indicate insufficient contractile stimulus for mitochondrial oxidative phosphorylation and result in greater *k* values (Adami et al. 2017).

$\dot{V}$O_2_*m* values derived from HHbR, HHbB, and StO_2_ were respectively fit by a monoexponential function according to Eq. 3:

3 $$y\left(t\right)={y}_{END}-A\times {e}^{-\frac{1}{\uptau }}$$

where *y*(t) =  $\dot{V}$O_2_*m* at a given time (t); *y*_*END*_ = $\dot{V}$O_2_*m* immediately after the cessation of the exercise; *A* = amplitude of the response; τ = exponential recovery rate constant (*k* = $\frac{1}{\uptau }$, expressed in min^−1^).

As previously indicated by Beever and colleagues (Beever et al. 2020), data were cleaned before curve fitting to remove invalid values or outliers.

One final value of *k* was reported for each variable even if two trial were performed. To do so, three different approaches were applied to average the trials:

Method 1: Overlap the data points from the two trials and then fit a single monoexponential curve;

Method 2: Average the pairs of data points from both trials according to their order of acquisition within each trial (i.e., average of the first data point of each trial, followed by the average of the second data point, etc.) and then fit a single monoexponential curve;

Method 3: Average the *k* values obtained from each trial using monoexponential fitting.

### Statistical analysis

Statistical analysis was conducted using SAS Studio 3.8 on SAS 9.4 (SAS Institute, Inc., Cary, NC, USA). We tested for differences in $\dot{V}$O_2max_ between CT and UT and local ATT between VL and TA using Welch’s *t* test and paired *t* test, respectively. We tested for differences between the averaging methods for *k* values obtained in HHbR, and between the different NIRS signals using Method 3 for *k* values estimated in VL muscle using linear mixed models. Specifically, we built a model that was equivalent to the one-way repeated measures ANOVA but we relaxed the assumption of sphericity by specifying an unstructured variance—covariance matrix for the residuals. We conducted multiple comparisons using paired *t* tests between the levels of the within-subjects factor whenever the *omnibus* test rejected the absence of an effect. Since the absence of evidence is not evidence of absence (Altman and Bland 1995), we also conducted pairwise comparisons using the two one-sided tests (TOST) procedure to test for equivalence (Schuirmann 1987) whenever the *omnibus* test failed to reject the absence of an effect. Unlike traditional null hypothesis significance testing, the TOST procedure allows formal equivalence testing by defining the upper and lower limits of practical significance (i.e. the equivalence margins) and testing the data against both limits in two one-sided tests, each conducted at the nominal alpha level. Equivalence can be established if both tests reject effects equal to or greater than the defined equivalence margins (Mazzolari et al. 2022). For the equivalence hypotheses, we set the equivalence margin at 5%, in agreement with the previous relevant literature (Sumner et al. 2020), and we applied the analytical approach recommended by Dixon and colleagues (2018) when none of the methods tested in the procedure (referred to as A and B) can be treated as an accurate reference. Briefly, we reformulated each hypothesis, originally expressed as a ratio of method A to B, as a linear combination of normally distributed random variables and tested the resulting deltas for each method against the nil (zero) effect. For a more comprehensive understanding of the statistical aspects of this approach, readers are encouraged to consult the original paper (Dixon et al. 2018). Regardless of the type of hypothesis, we controlled for multiple comparisons by applying the Bonferroni correction. We investigated the NIRS-derived muscle oxidative capacity in the VL and TA muscles in terms of fold of increase in $\dot{V}$O_2_*m* (Adami et al. 2017) obtained from HHbR during the knee extension exercise using a one-sample *t*-test against a prespecified cut-off value of 3 (Hanna et al. 2021). Additionally, we used the area under the density curve to calculate the probability for superiority, which is the probability that a person picked at random from the population will have a fold of increases in the VL and TA muscles greater than the cut-off. Finally, we tested for possible differences in *k* HHbR values between VL and TA at the same fitness level and within each muscle at the different fitness levels by constructing a linear mixed model, equivalent to a Split-Plot ANOVA for fixed effects. However, we allowed for differences in variances between CT and UT by specifying an unstructured variance–covariance matrix for the residuals within each fitness level and using the Satterthwaite approximation for degrees of freedom. Descriptive statistics are presented as mean ± SD and, where appropriate, also as median and interquartile range. Regardless of the distribution of the variables in our sample, we assumed asymptotic normality in most of our inferential models, given the size of our sample. The only exception was the last linear mixed model presented here, for which we assessed the normality of the residuals using a Q-Q plot. Inferential statistics are presented as mean difference (MD) or delta for method A (D_A_) or B (D_B_), test statistic with degrees of freedom, *p* value, and 95% confidence interval (CI). We set the alpha level to 0.05 for all hypotheses. In agreement with the statistical rationale of the TOST procedure, only the highest *p* value between the two one-sided tests and the associated statistics are reported. As future researchers may wish to test the robustness of our conclusions using different and perhaps more stringent equivalence limits or cut-off values, we have made the dataset related to those models available for this purpose, together with the SAS code and the workbook that performs all the pairwise calculations (https://osf.io/59asc/).

## Results

**Fig. 1:**
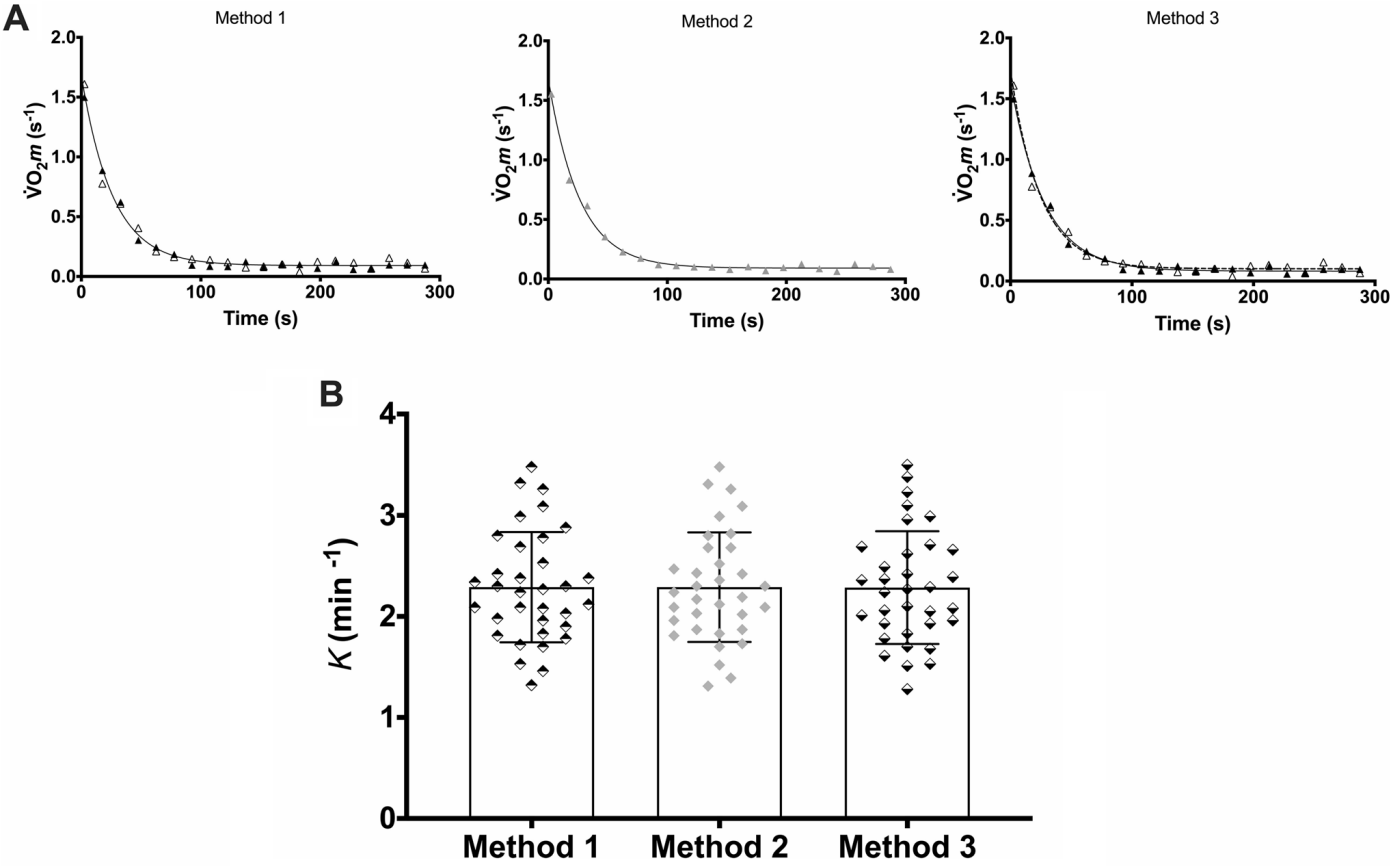
**A** Representative $\dot{V}$O_2_*m* values and respective monoexponential fittings of the three different approaches applied to average the trials: Method 1—Overlap the data points from trial 1 (black triangles) and trial 2 (white triangles) with a single monoexponential fitting (solid line); Method 2—Average the pairs of data points from trial 1 and trial 2 according to their order of acquisition within each trial (grey triangles) with single monoexponential fitting (solid line); Method 3 –data points from trial 1 (black triangles) with respective monoexponential fitting (solid line) and data points from trial 2 (white triangles) with respective monoexponential fitting (dashed line). **B** Mean values (± SD) of *k* determined with Method 1 (black and white rhombus), Method 2 (grey rhombus), and Method 3 (white and black rhombus)

The three averaging approaches identified in the methods were applied on $\dot{V}$O_2_*m* data obtained in VL using HHbR in 34 participants (Fig. 1). The *k* values obtained were 2.29 ± 0.55 min^−1^ for method, 2.29 ± 0.54 min^−1^ for method and 2.29 ± 0.56 min^−1^ for method 3. The *omnibus* test failed to find statistically significant differences between the average methods [*F* (2,33) = 0.09, *P* = 0.92]. However, the TOST procedure established equivalence in each pairwise comparison [method 1 *vs* method 2: D_A_ = 0.11 min^−1^,* t*(33) = 13.61, *P* < 0.001, 95% CI 0.10 to ∞; method 1 *vs* method 3: D_B_ =  − 0.12 min^−1^,* t*(33) =  − 7.56, *P* < 0.001, 95% CI − ∞ to − 0.08; method 2 *vs* method 3: D_B_ =  − 0.12 min^−1^,* t*(33) =  − 6.05, *P* < 0.001, 95% CI − ∞ to − 0.07] at the prespecified 5% margin.

Considering the obtained results, further analyses were performed adopting method 3.

**Fig. 2:**
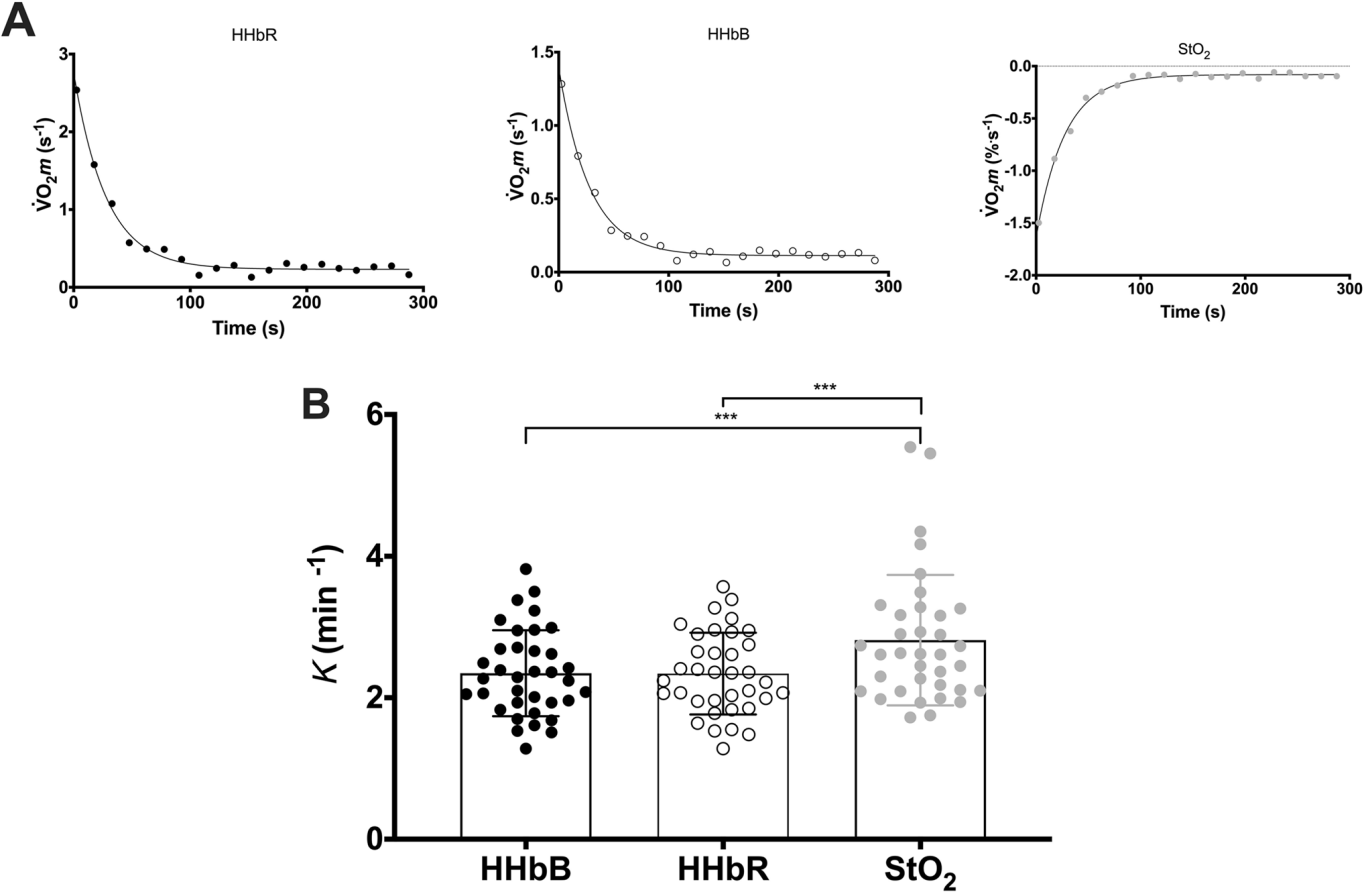
**A** Representative $\dot{V}$O_2_*m* values calculated on HHb data corrected for changes in blood volume following the Ryan et al. equation (HHbR, black circles), Beever et al. equation (HHbB, white circles), and StO_2_ (grey circles) with respective monoexponential fittings (solid lines). **B** Mean values (± SD) of *k* determined from HHbR (black circles), HHbB (white circles), and StO_2_ (grey circles). *** P < 0.001

The comparison between NIRS variables (HHb and StO_2_) and blood volume correction equations (HHbR and HHbB) were applied on $\dot{V}$O_2_*m* data obtained in VL (Fig. 2). The *k* values obtained were 2.35 ± 0.61 min^−1^ for HHbR, 2.34 ± 0.58 min^−1^ for HHbB, and 2.81 ± 0.92 min^−1^ for StO_2_. The *omnibus* test found statistically significant differences between the different NIRS signals [*F*(2,35) = 8.98, *P* < 0.001] with differences between *k* HHbR and *k* StO_2_ [MD =  − 0.47 min^−1^, *t*(35) =  − 4.18, *P* < 0.001, 95% CI − 0.75 to − 0.19] and between *k* HHbB and *k* StO_2_ [MD =  − 0.47 min^−1^, *t*(35) =  − 4.24, *P* < 0.001, 95% CI − 0.76 to − 0.19] and equivalence between *k* HHbR and *k* HHbB [D_A_ = 0.12 min^−1^, *t*(35) = 6.32, *P* < 0.001, 95% CI 0.08 to ∞] at the prespecified 5% margin.

**Fig. 3:**
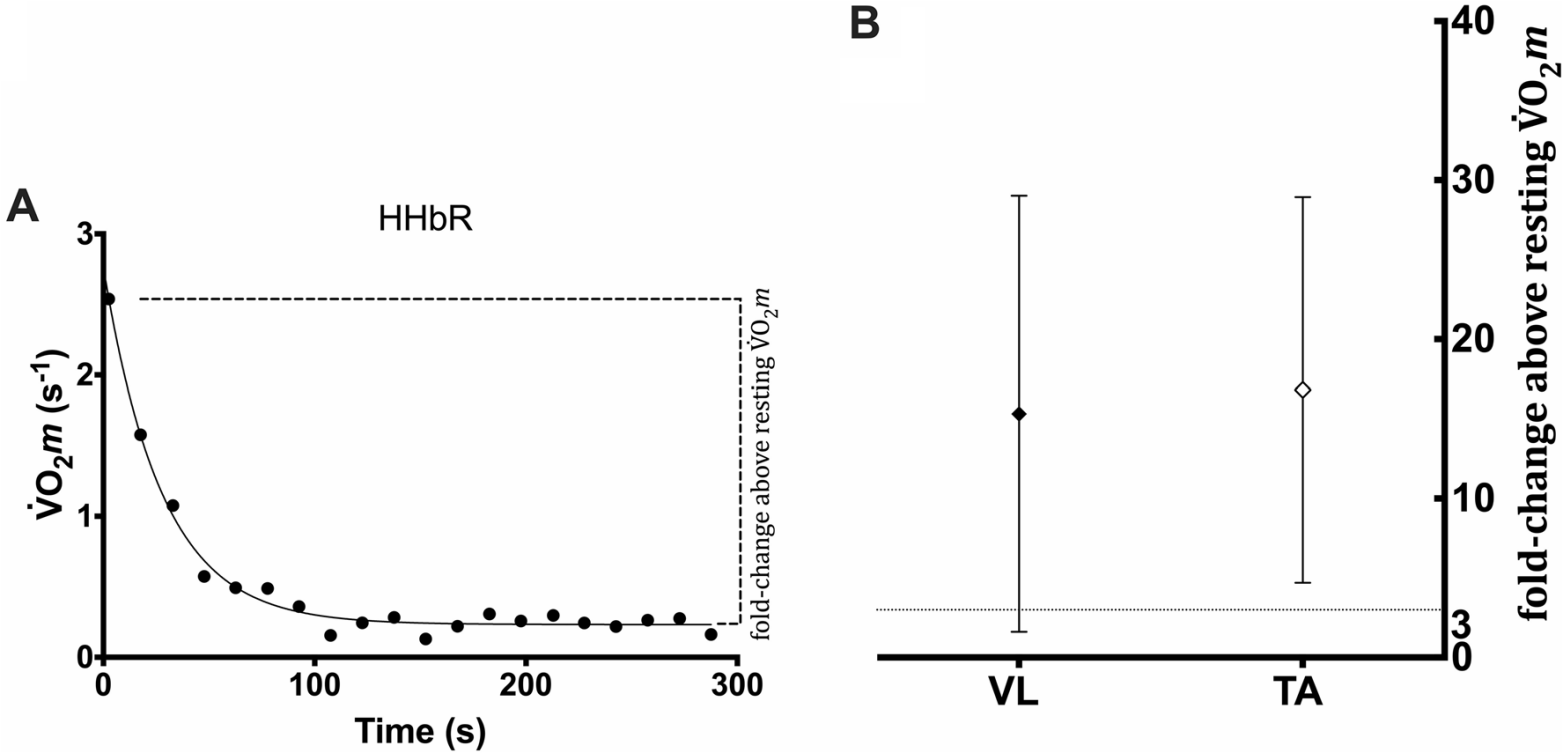
**A** Representative $\dot{V}$O_2_*m* values (solid circles) with the fold-change range (dashed bracket) estimated above the steady-state resting $\dot{V}$O_2_*m* range from the greatest V̇O_2_*m* recorded during the oxidative capacity test. **B** Mean values (± SD) of the fold-change above the steady-state resting $\dot{V}$O_2_*m* in VL (solid rhombus) and TA (empty rhombus) (B). Horizontal dotted line represents the cut-off of 3 proposed by Hanna et al. (2021)

Local ATT was lower in TA (0.45 ± 0.12 cm) compared to VL NIRS placement site (0.54 ± 0.14 cm) [MD =  − 0.09 cm, *t*(35) =  − 5.93, *P* < 0.001, 95% CI − 0.13 to − 0.05].The $\dot{V}$O_2_*m* recorded during the muscle oxidative capacity test and expressed as a fold-change above the steady-state resting $\dot{V}$O_2_*m* (Fig. 3A) were evaluated in both VL (15.3 ± 13.7 or 10.5 and 14.6 when expressed as mean ± SD or median and interquartile range, respectively) and TA (16.8 ± 12.1 or 12.4 and 18.0 when expressed as mean ± SD or median and interquartile range, respectively) muscle (Fig. 3B). The one-sample *t* test revealed a significant difference between both VL [MD = 12.3, *t*(35) = 5.39, *P* < 0.001, 95% CI 7.7–16.9] and TA [MD = 13.8, *t*(35) = 6.84, *P* < 0.001, 95% CI 9.7–17.9] and the prespecified cut off level of muscle activation (i.e., 3) in terms of folds of increase (Fig. 3B). These results translated into a probability for superiority of 81.3% (95% CI 71.1–88.8) and 86.9% (95% CI 78.6–92.6) against the cut-off for VL and TA, respectively.

**Fig. 4:**
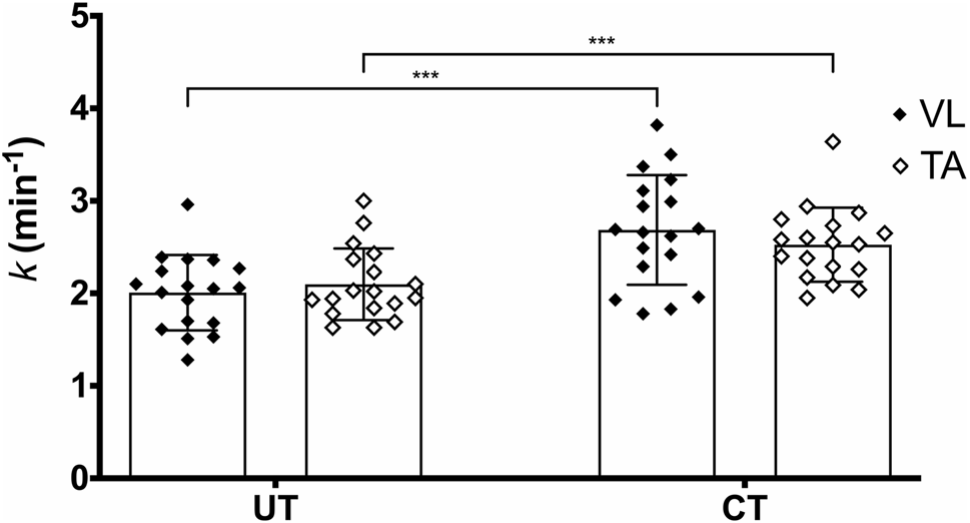
Mean values (± SD) of *k* in UT and CT participants in VL (solid rhombus) and TA (empty rhombus) muscles. ***P < 0.001

$\dot{V}$O_2max_ was greater in CT (58.2 ± 4.8 mL⋅kg^−1^⋅min^−1^) compared with UT participants (43.1 ± 4.9 mL⋅kg^−1^⋅min^−1^) [MD = 15.0 mL⋅kg^−1^⋅min^−1^, *t*(34.0) = 9.29, *P* < 0.001, 95% CI 10.8–19.3]. The *k* HHbR values obtained values were significantly greater in CT compared to UT in both VL [(MD = 0.68 min^−1^, *t*(30.1) = 4.00, *P* = 0.002, 95% CI 0.23–1.13] and TA [(MD = 0.43 min^−1^, *t*(34.0) = 3.27, *P* = 0.01, 95% CI 0.08–0.77] (Fig. 4), whereas no statistically significant differences were detected between VL and TA within the same group [CT: MD = 0.16 min^−1^, *t*(17) = 0.99, *P* = 1.00, 95% CI − 0.29 to 0.61; UT: MD =  − 0.09 min^−1^, *t*(17) =  − 0.77, *P* = 1.00, 95% CI − 0.42 to 0.24] (Fig. 4).

## Discussion

The present study investigated how different analysis strategies proposed in the literature might affect the evaluation of muscle oxidative capacity using NIRS-derived outcomes. The main findings were that, in a large group of healthy females and males of different fitness levels: (I) different averaging methods for multiple trials led to similar *k* values; (II) although different blood volume correction strategies for HHb lead to similar *k* values, the HHb and StO_2_ signals resulted in different *k* response; (III) Higher *k* values were detected in CT than UT, regardless of the evaluated muscle (VL or TA).

The originally proposed protocol for the NIRS derived muscle oxidative capacity has been adapted over time to suit different projects and research settings, as highlighted by Adami and Rossiter (Adami and Rossiter 2018), who reported *k* values for muscle oxidative capacity from 19 studies and almost as many different protocols. Whereas this flexibility in the application of the testing protocols for NIRS-derived muscle oxidative capacity evaluation is practical, it also poses challenges when comparing results within the literature. To circumvent this issue, recommendations have been made to ensure successful evaluations of muscle oxidative capacity such as: averaging at least two trials for each participant, correcting for changes in blood volume when the rate of increase in HHb is used for the evaluation, using adequate exercise/contractions intensities and durations to stimulate oxidative phosphorylation without restraining blood flow (Barstow 2019). Despite the relevance of these recommendations, no study has systematically evaluated their validity.

*Averaging approach*: While there is agreement in that averaging two or more trials reduces variability enhancing the signal-to-noise ratio, how this average should be performed or how different averaging methods might affect the main outcome of the evaluation remains unclear (e.g. Zuccarelli et al. 2020; Pilotto et al. 2022). In the present study, we assessed the impact of three different averaging methods, for the two trials performed, on the final *k* value. Our results demonstrated that not only were the three averaging methods not different from each other but they also proved equivalent within a reasonably narrow margin. This information is important as it allows researchers to confidently adopt any of these averaging methods without being concerned to affect the final outcome.

*NIRS signal to use for the evaluation*: Different NIRS variables have been utilized to calculate the slope to estimate $\dot{V}$O_2_*m* including O_2_Hb (Motobe et al. 2004), HHb (Ryan et al. 2014), Hb_difference_ (Hb_difference_ = O_2_Hb – HHb) (Southern et al. 2014), and StO_2_ or tissue saturation index (TSI) (Adami et al. 2017). In particular, HHb has been utilized as the preferred index of deoxygenation over O_2_Hb and Hb_difference_, because it is relatively insensitive to blood volume changes and has been demonstrated to correlate well with other variables related to fractional O_2_ extraction (Grassi and Quaresima 2016). Moreover, possible issues related to blood volume changes have been solved by utilizing, for values during occlusions, blood volume correction equations as the ones proposed by Ryan et al. (Ryan et al. 2012) and Beever et al. (Beever et al. 2020). Alternatively, StO_2_ has been commonly utilized since this signal intrinsically considers changes in total hemoglobin (tHb = O_2_Hb + HHb) often used as an index of blood volume in the investigated tissue (StO_2_ = $O_{2} Hb/tHb$) [see Quaresima (Chung et al. 2018)]. In our investigation, we reported no practically-relevant differences between blood volume correction equations (HHbR *vs* HHbB) in the determination of *k* values, and the two methods resulted equivalent in a sample of females and males of different fitness levels. As already suggested by Beever and colleagues, differences between these two blood volume correction equations are small unless the change in tHb is large or $\frac{\left|{{O}_{2}Hb}_{(t)}\right|}{\left(\left|{{O}_{2}Hb}_{(t)}\right|+\left|{HHb}_{(t)}\right|\right)}$ deviates considerably from 50% (Beever et al. 2020). On the contrary, *k* values were significantly greater when determined using StO_2_ compared to HHb, with a difference of ~ 0.5 min^−1^ (from 0.2 to 0.8 min^−1^ when considering the 95% CI). These results were surprising as both variables are indicative of fractional O_2_ extraction and consider blood volume changes. Importantly, our results highlight the importance of comparing *k* values derived from the same NIRS variables. A difference of 0.5 min^−1^ on an average *k* value of 2.5 min^−1^ as found in our young healthy individuals represent a difference of 20% which can be significant in studies evaluating training intervention or training status (Lagerwaard et al. 2021) as well as detraining (Zuccarelli et al. 2021) or pathologies (Erickson et al. 2015). To give a practical example, if UT *k* values were determined with StO_2_ while CT *k* values were determined with HHb we would have had average values of 2.45 and 2.69 min^−1^, that are not significantly different between each other.

*Adipose tissue thickness and intensity of muscle contraction*: Independent of the selected NIRS technique used for evaluation, a factor that universally affects the signal strength is the ATT over the muscle of interest. In fact, greater ATT will reduce the underlying skeletal muscle interrogated by the NIRS signals, resulting in reduced absorption of NIR light by muscle chromophores, ultimately affecting the quality of the outcomes. However, it is possible to overcome this issue by making sure every participant has an ATT no greater than the penetration depth of the NIR light (i.e., half the distance between the NIRS device source and detector, in this specific investigation 2 cm). All our participants had an ATT for both VL and TA no greater than 1 cm (VL 0.14–0.54 cm, TA 0.12–0.45 cm). However, it is not always possible to select participants with ATT values no greater than 1/4 the longest source-detector distance, without compromising the selected population characteristics, as previously suggested (Barstow 2019). To overcome the possible ATT issue, in this project we simultaneously evaluated VL muscle and TA muscle, since the latter usually shows lower ATT, as further confirmed by ATT values in our participants.

The challenge of simultaneous testing of two different muscles resided in ensuring a proper activation of both muscles, to stimulate oxidative phosphorylation without restraining blood flow which is pivotal for successful data collection. Insufficient intensity of exercise, and therefore, a limited increase in $\dot{V}$O_2_*m*, would impair measurement accuracy and reliability. Ryan and colleagues demonstrated that exercise type (active exercise or electrical stimulation) or intensity (frequency of contraction or stimulations) do not need to be strictly controlled as long as $\dot{V}$O_2_*m* increases sufficiently (Ryan et al. 2013a). Related to this last assumption, it has been suggested that the intensity of exercise should be carefully considered for the assessment of muscle oxidative capacity by NIRS [see Porcelli et al. (Chung et al. 2018)] by measuring force output or external work rate (Zuccarelli et al. 2020), or at least estimate the $\dot{V}$O_2_*m* increase during exercise, with respect to the resting baseline. In this study, we evaluated the increment of $\dot{V}$O_2_*m* induced by exercise in both VL and TA, following a procedure described by Adami and colleagues (Adami et al. 2017). The change in $\dot{V}$O_2_*m* induced by the exercise was estimated from the greatest $\dot{V}$O_2_*m* recorded during the muscle oxidative capacity test and expressed as a fold-change above the steady-state resting $\dot{V}$O_2_*m* (measured at the end of the muscle oxidative capacity test). Using this approach, we observed that the increases were consistently greater than a cut off value of threefold increase proposed by Hanna and colleagues (Hanna et al. 2021). Even though our leg extension exercise protocol targeted mainly VL muscles, the metabolic perturbation induced by the exercise resulted in sufficient increases in $\dot{V}$O_2_*m* in both the VL and the TA muscles, which were sufficient for activating mitochondrial oxidative enzymes without impairing oxygen delivery.

The direct comparison between VL and TA *k* values within the same fitness level group did not reveal any statistically significant difference in the *k* values, but differences were found when comparisons for the same muscle were performed between CT and UT. Our data for VL are in line with previously reported values for healthy inactive and endurance trained individuals (Brizendine et al. 2013). Additionally, our data also detected training related differences in NIRS-derived muscle oxidative capacity in the TA, which indicates that performing this evaluation on a leg muscle that is typically characterized by lower levels of ATT (i.e., TA) and that is easily accessible results in similar responses as compared to the evaluations performed in the VL. Concerns can be raised regarding the different fiber composition as well as capillarization between the VL and TA muscles, since in the general population the VL muscle has a greater percentage of type II fibers compared to type I (Staron et al. 2000; Horwath et al. 2021) while the TA has a higher percentage of type I fibers (Jaworowski et al. 2002; Porter et al. 2002; Holmbäck et al. 2003). These concerns can be partially overcome by the intrinsic characteristics of NIRS technology. That is, commercially available NIRS instruments can interrogate only a relatively small (2–6 cm^3^) and superficial volume of skeletal muscle tissue (Grassi and Quaresima 2016), which might not represent the whole muscle accurately considering the heterogeneity of muscle blood flow and the uneven spatial distribution of fiber types, with a more predominant content of type II fibers at the surface of the muscle and type I in deeper regions (Chung et al. 2018).

*Experimental considerations*: A point of consideration in this study is the selected modality and intensity of exercise, which could potentially impair measurement accuracy and reliability. One assumption of NIRS-derived skeletal muscle oxidative capacity evaluation is that brief muscle contractions maximally activate mitochondrial oxidative enzymes, according to the first-order relationship between phosphocreatine dynamics and ATP resynthesis by oxidative phosphorylation (Wüst et al. 2011, 2013). Ryan and colleagues demonstrated that exercise type (active exercise or electrical stimulation) or intensity (frequency of contraction or stimulations and current intensity of electrical stimulation) do not need to be strictly controlled as long as $\dot{V}$O_2_*m* increases sufficiently (eight–tenfold above resting), which can be achieved with 10–20 s of low- to moderate intensity exercise (Ryan et al. 2013a). In this study, we observed that the increases were for VL and TA muscles on average over 15-fold of increase above resting, which is in line with previous investigations. Whereas the exercise modality, duration, and intensity utilized in this project looked adequate to activate mitochondrial oxidative enzymes without limiting oxygen availability, we cannot demonstrate that the exercise intensity was inducing the same metabolic disturbance in both chronically trained and untrained participants. Future studies are necessary to further investigate the impact of modality and intensity of exercise for the investigation of muscle oxidative capacity in different groups.

## Conclusion

Our study provided novel experimental information on how different approaches for data processing can affect NIRS-derived evaluations of muscle oxidative capacity, showing that different methods for averaging trials can be used interchangeably and different blood volume corrections do not impact *k* values, but different NIRS variables lead to different *k* values estimation. Moreover, we demonstrated that fitness level differeces can be detected in both vastus lateralis and tibialis anterior in participants of different fitness level. Therefore, even though there are some factors to consider when comparing results from different studies, NIRS-derived evaluation of muscle oxidative capacity is, for the most part, a robust test that allow for consistent outcomes.

### Supplementary Information

Below is the link to the electronic supplementary material.

Supplementary file1 (XLSX 110 KB)

## Data Availability

The datasets generated during and/or analysed during the current study are available as supplementary material.

## Abbreviations

*A*: Amplitude of the response
ANOVA: Analysis of variance
ATT: Adipose tissue thickness
CT: Chronically trained
HHB: Deoxygenated hemoglobin
HHbB: Deoxygenated hemoglobin with Beever’s blood volume correction
HHbR: Deoxygenated hemoglobin with Ryan’s blood volume correction
*k*: Rate constant
NIRS: Near-infrared spectroscopy
O_2_Hb: Oxygenated hemoglobin
StO_2_: Oxygen saturation
t: Time
τ: Time constant
TA: Tibialis anterior
tHb: Total hemoglobin
TOST: Two one-sided tests
UT: Untrained
VL: Vastus lateralis
$\dot{V}$O_2_*m*: Muscle oxigen consumption
$\dot{V}$O_2max_: Maximal oxigen consumption
